# A prospective study of a rapid adaptive version of the Boston Naming Test for Alzheimer’s disease

**DOI:** 10.1002/alz.085152

**Published:** 2025-01-03

**Authors:** Julia H. Cho, Siva Muthupalaniappan, Thomas A. Giauque, Maurice Smith, Daniel Z. Press

**Affiliations:** ^1^ Harvard Medical School, Boston, MA USA; ^2^ UMass Chan Medical School, Worcester, MA USA; ^3^ Harvard University School of Enginering and Applied Sciences, Cambridge, MA USA; ^4^ Department of Neurology, Harvard Medical School, Boston, MA USA; ^5^ Berenson‐Allen Center for Noninvasive Brain Stimulation, Beth Israel Deaconess Medical Center and Harvard Medical School, Boston, MA USA

## Abstract

**Background:**

Cognitive tests of naming ability have been shown to have diagnostic and prognostic utility in both mild cognitive impairment (MCI) and Alzheimer’s disease (AD; Taler & Phillips, 2008). The Boston Naming Test (BNT) is the most common naming test, which consists of 60 black‐and‐white drawings and takes 20‐30 minutes to administer. Retrospective analysis has shown that administering the BNT in an adaptive fashion could result in a comparable measure of the patient’s naming ability in only 8 items instead of 60. A prospective administration of this adaptive naming test (ANT) was necessary to assess its utility in clinical practice.

**Method:**

Using item response theory (IRT) and published information about each BNT item’s difficulty and discriminability (Pedraza et al., 2011), we created an algorithm to administer the traditional BNT in an adaptive format. Patients were recruited from the Cognitive Neurology clinic at Beth Israel Deaconess Medical Center. Participants completed both a 30‐item traditional BNT (either odd or even‐numbered items) and a 10‐item ANT (selected from the remaining set of 30 items). Randomization was used to decide which test would be first, and which would use the odd or even‐numbered items. Z‐scores were calculated for BNT scores using normative data from Katsumata et al. (2015). ANT scores were calculated according to IRT principles (Pedraza et al., 2011). Total administration time from the two tests were compared using paired t‐tests, and descriptive statistics results are presented as mean±SD.

**Result:**

We have begun testing patients with the traditional and adaptive forms of the BNT (n = 7). 28% of patients have MCI and 57% have mild‐to‐moderate AD. Average administration times were 611±222 seconds for the BNT and 59±13 seconds for the ANT (difference = 552 seconds, p = 0.0013). After removing one outlier, the standardized BNT scores and ANT scores exhibit a linear relationship with an r‐squared value of 0.977.

**Conclusion:**

This prospective administration of the ANT confirms prior research that an adaptive BNT performs significantly faster while still giving a reliable measure of one’s naming ability. We expect ongoing data collection to further strengthen the robustness of our findings.

